# IL-11 in fibrostenotic Crohn’s disease: historical agonism, the selective-inhibition hypothesis, and evidence thresholds for translation

**DOI:** 10.3389/fphar.2026.1951110

**Published:** 2026-10-16

**Authors:** He Chen, Shuang Liang

**Affiliations:** The Fourth People’s Hospital of Shenyang, Shenyang, Liaoning, China

**Keywords:** biomarker development, Crohn’s disease, fibrostenosis, IL-11, IL11RA, intestinal fibrosis, receptor pharmacology, stromal cells

## Abstract

Effective medical treatment for fibrostenotic Crohn’s disease remains a major unmet clinical need. This narrative review examines whether high stromal interleukin-11 (IL-11) identifies a pharmacologically reversible dependency in established fibrostenotic disease or mainly marks advanced remodeling. Historical trials found schedule-dependent activity of recombinant human IL-11, but an active-comparator trial showed inferior remission induction compared with prednisolone. In mice, *Il11* or *Il11ra1* deficiency worsened epithelial apoptosis and acute dextran sulfate sodium (DSS) colitis. By contrast, inducible *Il11* deletion during repeated DSS injury reduced collagen deposition and hydroxyproline with broadly similar inflammatory trajectories. These contrasting findings support context-dependent effects across acute injury and chronic remodeling. Human tissue, serum and treatment-response studies associate IL-11 with fibrostenosis and stromal activation, but do not establish that an IL-11-high lesion responds to selective inhibition. Differences in fibroblast state, tissue compartment and treatment timing could therefore influence both efficacy and harm. Early LASN01 studies outside inflammatory bowel disease support systemic target engagement and preliminary tolerability; intestinal efficacy remains untested. The decisive next test is selective inhibition after a structural lesion is documented, with verified intestinal exposure and separate structural, inflammatory and repair outcomes. This would distinguish reduced collagen accumulation during disease development from reversal of an established Crohn’s stricture.

## 1 Introduction

Effective medical treatment for fibrostenotic Crohn’s disease remains a major unmet clinical need. Anti-inflammatory therapies can improve inflammatory activity, but established strictures often still require endoscopic balloon dilation or surgery. Importantly, a stricture is not synonymous with collagen accumulation: it is a structural lesion in which inflammation, edema, extracellular-matrix (ECM) deposition, smooth-muscle remodeling, altered neural and vascular biology, and mesenteric changes may coexist. This complexity is one reason why a pathway can be biologically credible while remaining an unproven drug target (Rieder et al., 2017; Yoo et al., 2020; Li et al., 2019).

IL-11 is a member of the IL-6 cytokine family. It binds IL-11Rα and recruits the shared signal transducer gp130, activating signaling that depends on the responding cell and experimental conditions. IL-11Rα expression helps determine responsiveness, whereas gp130 is shared with other cytokine pathways (Barton et al., 2000; Sentosa et al., 2025). Thus, inhibition of IL-11, IL-11Rα, gp130 or downstream kinases tests different biological questions.

Historical studies tested recombinant human IL-11 (rhIL-11) in active inflammatory Crohn’s disease, whereas current inhibitor proposals focus on intestinal fibrosis. Two placebo-controlled trials reported exploratory activity, but rhIL-11 was inferior to prednisolone for remission induction (Sands et al., 1999; Sands et al., 2002; Herrlinger et al., 2006). Subsequent studies linked IL-11 to fibroblast activation and matrix production (Schafer et al., 2017; Ng et al., 2019). Intestinal experiments now include stromal overexpression, acute deficiency and inducible deletion during chronic injury (Lim et al., 2020; Nishina et al., 2023; Pokatayev et al., 2026). Their different outcomes require interpretation by disease stage, species and intervention.

Broad multi-organ reviews have synthesized IL-11 biology and fibrosis (Cook, 2023; O’Reilly, 2023). By contrast, the purpose of the present review is to test the pharmacological logic and translation thresholds of the selective-inhibition hypothesis specifically in fibrostenotic Crohn’s disease.

We deliberately focus on a narrower question: does an IL-11-high stromal state identify an ongoing, pharmacologically reversible dependency in established fibrostenotic Crohn’s disease, or does it mainly mark advanced remodeling? Here, dependency means that an established lesion continues to rely on IL-11 activity, such that selective inhibition could improve bowel-wall structure and function. We first review the historical trials and receptor pharmacology, then examine bowel-wall pathology, intestinal experiments and human studies. The final sections consider biomarkers, safety and the studies needed to test this hypothesis. ‘IL-11-high’ denotes increased stromal *IL11* expression or IL-11 production relative to a specified comparator; its research definition is developed in Section 6.5. Figure 1 summarizes the historical evidence and the remaining tests of pharmacological reversibility. The evidence principles guiding our interpretation are summarized in Box 1.

**FIGURE 1:**
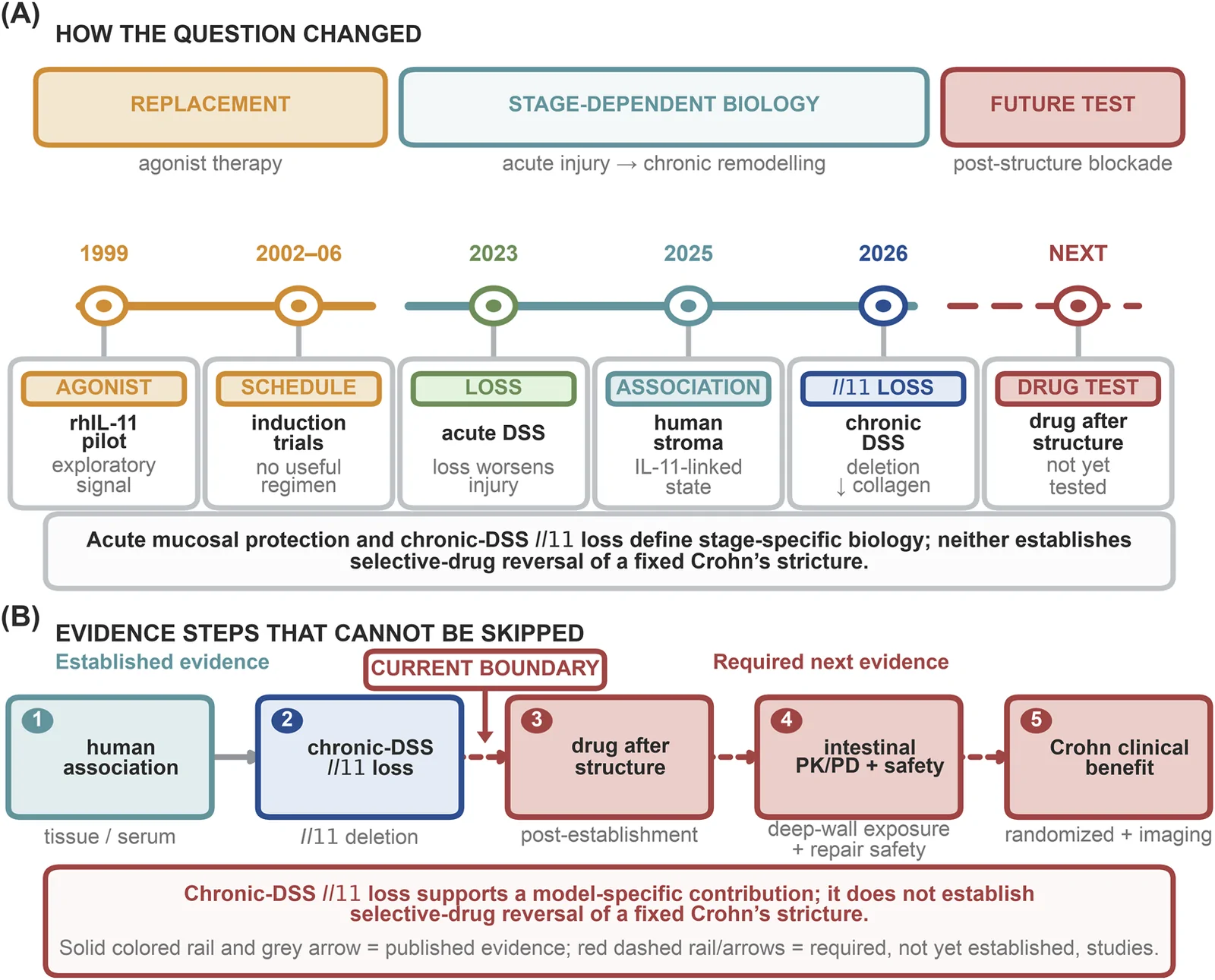
Timeline of the IL-11 pharmacological reversal and evidence gates for target advancement **(A)** Recombinant human interleukin-11 (rhIL-11) agonist trials, acute dextran sulfate sodium (DSS) loss-of-function data, human stromal association and chronic-DSS *Il11* loss-of-function evidence define the published trajectory. The dashed red transition marks selective pharmacology initiated after structural disease is demonstrable as an untested next step. **(B)** Human association and chronic-DSS loss-of-function evidence precede three unresolved gates: post-establishment drug intervention, intestinal pharmacokinetics/pharmacodynamics (PK/PD) with safety assessment and imaging-anchored Crohn’s benefit. The grey connector links the two published evidence classes; dashed red connectors and red boxes denote required evidence that has not yet been established. The figure does not claim selective drug-mediated reversal of an established stricture.

Box 1 **Four evidence principles used in this review.**

- Association is not causal dependency. Tissue expression, serum concentration and treatment-response correlations can motivate biomarker work but do not establish therapeutic benefit.
- Sufficiency, genetic contribution and pharmacological reversibility are distinct. A gain-of-function model can show that a pathway can generate pathology. Chronic-DSS loss-of-function can show a model-specific contribution. Neither establishes drug-mediated reversal of an established human stricture.
- Selective pharmacology matters. Changes after gp130, ERK or JAK/STAT inhibition cannot identify IL-11 as the responsible upstream ligand.
- Cross-organ efficacy is not intestinal validation. Extraintestinal work can support tractability, mechanism and safety hypotheses, but cannot establish reversal of a Crohn’s stricture.

We distinguish diagnostic, prognostic, predictive and pharmacodynamic biomarkers according to their intended use. Their specific validation requirements are discussed in Section 7.

## 2 Literature approach and evidence grading

This is a critical narrative review, not a systematic review or meta-analysis. PubMed was searched through 28 July 2026, with a targeted literature update on 13 September 2026; Crossref was used to verify bibliographic metadata. Search concepts included IL-11 and its receptors, Crohn’s disease, intestinal fibrosis, strictures, stromal cells, receptor pharmacology and clinical development. Search queries and the scope of the targeted update are provided in Supplementary Methods.

We prioritized randomized human studies; direct human intestinal studies with stated tissue source; paired strictured/non-strictured material; spatial or single-cell datasets with independent validation; genetic or selective pharmacological perturbation; and receptor studies that specified species, ligand and assay context. Studies were categorized as: (i) direct intestinal human evidence, (ii) direct intestinal experimental evidence, (iii) receptor or species pharmacology, (iv) cross-organ pharmacological plausibility, and (v) safety or clinical-development evidence. When a report reused a public cohort or analyzed a related subset, it was treated as non-independent support. No pooling was performed because model systems, interventions and endpoints are too heterogeneous.

## 3 Historical recombinant IL-11 development in Crohn’s disease

### 3.1 Preclinical rationale for recombinant IL-11

Early studies linked IL-11 to hematopoiesis, mucosal recovery and reduced inflammatory signaling. These observations motivated cytokine administration in active Crohn’s disease, before current distinctions between inflammatory and structural endpoints were routinely applied.

The original protective evidence was heterogeneous and largely concerned acute injury rather than chronic transmural remodeling. In mice given combined chemotherapy and radiation, IL-11 increased crypt-cell proliferation, accelerated small-intestinal mucosal recovery and improved survival; a follow-up study in 78 C3H/HeJ mice linked this response to less crypt apoptosis and faster villus recovery (Du et al., 1994; Orazi et al., 1996). In a separate report, twice-daily subcutaneous rhIL-11 reduced injury in a hamster oral-mucositis model and in rat acute-colonic-injury and HLA-B27 transgenic colitis models (Keith et al., 1994). rhIL-11 also reduced inflammatory mediator production and NF-κB nuclear activity in activated macrophages (Trepicchio et al., 1997). These studies explain why agonist development appeared reasonable; they do not demonstrate a therapeutic effect on established fibrostenotic Crohn’s disease.

Supplementary Table S2 specifies the species, interventions and endpoints of these studies.

### 3.2 Randomized trials in active Crohn’s disease

In the 1999 multicenter dose-escalation trial, 76 participants with active Crohn’s disease received placebo or subcutaneous rhIL-11 over 3 weeks. The 16 μg/kg/week regimens produced the highest exploratory response rates (33%–42% in the relevant small dose groups *versus* 7% with placebo), while platelet responses demonstrated dose- and schedule-dependent biology (Sands et al., 1999). The study was not designed to establish durable efficacy or anti-fibrotic benefit. It did, however, justify a larger study.

The 2002 randomized study included 148 evaluable participants with mildly to moderately active disease. In the once-weekly 15 μg/kg arm, remission occurred in 36.7% of participants *versus* 16.3% with placebo; the primary continuous Crohn’s Disease Activity Index (CDAI) endpoint showed a favorable but non-significant trend, and the twice-weekly regimen did not replicate the remission signal. Injection-site reactions, headache, edema and platelet increases were more common in the twice-weekly group (Sands et al., 2002). This pattern is compatible with a small, schedule-sensitive clinical signal, not with a robustly validated induction treatment.

The 2006 double-blind trial compared rhIL-11 with prednisolone in 51 patients. Intention-to-treat remission at week 4 was 4% *versus* 46%; at week 6, it was 19% *versus* 50%, respectively. Fever, rash, arthralgia or arthritis, nausea or vomiting, and headache occurred in rhIL-11-treated participants (Herrlinger et al., 2006). Thus, systemic rhIL-11 was not a useful alternative to corticosteroid induction in this study. None of the three trials evaluated prevention of stricture progression or reversal of an established stricture.

### 3.3 Interpretation of the historical trials

All three Crohn’s trials administered human IL-11 to human participants. Their clinical outcomes therefore cannot be attributed to human–mouse ligand mismatch. Species-dependent pharmacology remains relevant to the rodent experiments used to explain those outcomes.

These trials assessed short-term inflammatory activity, mainly using the CDAI. They did not select patients by imaging-defined fibrostenosis or measure bowel-wall mechanics. Their results neither validate IL-11 inhibition nor exclude a different effect in a different disease stage. Historical agonist exposure also cannot establish the safety of prolonged ligand or receptor blockade, which requires separate evaluation.

## 4 IL-11 receptor pharmacology and species effects

### 4.1 Receptor assembly and intervention strategies

IL-11 first binds IL-11Rα and then recruits the shared signal transducer gp130. Classical biochemical studies described a high-affinity hexameric complex containing two molecules each of IL-11, IL-11Rα and gp130; distinct ligand surfaces participate in receptor and gp130 engagement (Barton et al., 1999; Barton et al., 2000). More recent structural work provides a human complex-level view and links receptor architecture to disease-associated receptor variants (Sentosa et al., 2025). IL-11Rα expression, unlike broadly expressed gp130, helps define cell responsiveness; receptor-domain organization, ligand-binding domain and receptor-stalk length influence classical signaling (Schleinkofer et al., 2001; Nitz et al., 2015; Lokau and Garbers, 2018).

Ligand neutralization, IL-11Rα blockade and downstream inhibition differ in selectivity and pharmacokinetics. Anti-ligand antibodies can change total circulating IL-11 through complex formation and altered clearance (Mager and Jusko, 2001; Yang and Quarmby, 2011). Receptor antibodies have distinct occupancy, internalization and tissue-distribution properties. Because gp130 and JAK/STAT signaling are shared, their inhibition cannot establish IL-11-specific activity. Total serum IL-11 or phosphorylated STAT3 alone is therefore an insufficient intestinal target-engagement measure.

Pharmacokinetic/pharmacodynamic (PK/PD) studies should connect intestinal exposure with receptor occupancy or validated proximal signaling, followed by matrix and functional outcomes. Free and total ligand require separate assay definitions. Early signaling and later fibroblast responses may differ temporally (Widjaja et al., 2021).

Plasma exposure may not predict drug concentrations in a thickened, poorly perfused bowel segment. Blood-cell occupancy also cannot establish receptor occupancy in submucosal fibroblasts or muscularis propria cells. Ligand and receptor blockade should therefore be compared in primary human intestinal cells and multicellular preparations at measured exposures.

The contribution of soluble IL-11Rα signaling to Crohn’s strictures remains uncertain. Engineered-cell results and receptor-domain studies do not establish its importance within individual bowel-wall layers (Lokau and Garbers, 2018). Proposed soluble-receptor mechanisms require measurements and functional validation in intestinal tissue.

### 4.2 Species-dependent assays and conflicting fibroblast results

Studies using overlapping investigators and experimental platforms reported different responses to recombinant human and murine IL-11 in mouse fibroblasts. These included STAT3 phosphorylation in *Il11ra1*-null cells at high human-ligand concentrations (Widjaja et al., 2021). The findings warrant species-matched ligands, receptor controls and concentration-response measurements. They should not be generalized to every historical experiment or treated as independent replication across laboratories.

Discordant findings also occur in human systems. IL-11-dependent responses have been reported in dermal and other fibroblasts (Adami et al., 2021; Widjaja et al., 2021). However, an independent lung-cell study found that IL-11 blockade did not prevent transforming growth factor beta (TGF-β)-induced profibrotic responses (Tan et al., 2024). Cell origin, baseline activation, culture conditions and endpoint selection may explain some differences. IL-11 may contribute strongly in selected settings without being required for all TGF-β-driven fibrosis. Potential explanations for these divergent findings are summarized in Box 2.

BOX 2 **Competing explanations for divergent IL-11 results.**

- **Ligand–species mismatch:** human and mouse ligands, receptors and neutralizing reagents may not be functionally equivalent in a given assay.
- **Exposure design:** concentration, pulse *versus* sustained exposure, and acute *versus* chronic timing can change measured outputs.
- **Cellular context:** receptor abundance, cell composition, matrix stiffness and inflammatory background may determine whether IL-11 is consequential.
- **Assay and reagent limitations:** antibody epitope, cross-reactivity, endotoxin control, receptor-null/rescue controls, and reliance on one phospho-protein can generate misleading certainty.
- **True biological heterogeneity:** IL-11 could be causal in particular stromal states without being the obligatory mediator of all TGF-β-dependent fibrosis.

These alternatives can be tested with matched ligands, selective inhibitors and genetic controls in the same intestinal preparation. Replication should measure signaling, protein translation and matrix outputs over time, rather than relying on a single downstream marker.

## 5 Bowel-wall pathology and assessment of Crohn’s strictures

### 5.1 Fibrosis and other contributors to narrowing

Crohn’s strictures comprise anatomic narrowing, bowel-wall thickening and pre-stenotic dilation, but their cellular composition varies across patients and along a lesion. Fibrosis reflects ECM accumulation and cross-linking; smooth-muscle hypertrophy and hyperplasia can independently contribute to luminal narrowing; myofibroblasts, pericytes, vascular and neural compartments, immune infiltrates, edema and mesenteric creeping fat participate in a spatially organized remodeling process (Li et al., 2019; Alfredsson and Wick, 2020; Veisman et al., 2024). Activated muscularis propria cells can also create an ECM niche that promotes preadipocyte migration and creeping-fat formation, illustrating that the stricture phenotype extends beyond a fibroblast-only model (Mao et al., 2022).

An IL-11-dependent fibroblast response could account for only part of a stricture. Reduced collagen expression may leave smooth-muscle expansion, stiffness and luminal narrowing unchanged. Conversely, reduced edema may improve imaging without indicating matrix regression. Structural and inflammatory outcomes should therefore be measured separately.

Ileal, ileocolonic, anastomotic and colonic strictures differ in anatomy, prior injury and access for endoscopic assessment. Lesion length, upstream dilation and penetrating complications also influence the opportunity for medical treatment. These features should be recorded when comparing molecular profiles or treatment outcomes.

Inflammation and fibrosis often coexist rather than following a fixed sequence. Active inflammation can cause edema, while advanced fibromuscular narrowing can remain symptomatic despite low mucosal inflammatory activity. Clinical remission or reduced fecal calprotectin therefore does not establish regression of fibrosis.

Neural and vascular remodeling may also affect motility, tissue perfusion and drug delivery. These components strengthen the rationale for measuring bowel function alongside matrix responses (Li et al., 2019; Alfredsson and Wick, 2020).

Mesenteric signals may amplify intestinal fibroblast activation. Chen et al. reported enrichment of TINAGL1 in mesenteric adipose-derived exosomes from Crohn’s surgical specimens, using colorectal-cancer surgical specimens as controls. Disease-associated exosomes activated primary intestinal fibroblasts, while exosome administration or recombinant TINAGL1 increased fibrosis during repeated dinitrobenzene sulfonic acid (DNBS) injury in mice. Protein-interaction and signaling experiments implicated TGF-β/SMAD4 signaling (Chen et al., 2025a). This provides adjacent mechanistic evidence for mesenteric–fibroblast communication. Its dependence on TINAGL1, connection to IL-11 and persistence after stricture formation remain to be tested.

### 5.2 Tissue compartments and sampling limitations

Mucosal biopsies are accessible but often do not sample the submucosa or muscularis propria, where key fibromuscular changes reside. Surgical specimens provide full-thickness information but are enriched for severe, treatment-refractory disease and are usually cross-sectional. Existing histopathology scores have important limitations: a systematic review found no validated, reproducible reference index that fully captures both fibrosis and muscular hypertrophy in Crohn’s stenosis (Gordon et al., 2020). Therefore, a single mucosal transcript or collagen stain cannot be considered an adequate proxy for a transmural stricture.

Microdissected submucosa and muscularis propria from strictured ileum show different matrix, immune, metabolic and muscle-associated proteomic changes (Alfredsson et al., 2026). Spatial transcriptomics likewise identifies localized myoid-cell expansion and fibroblast populations expressing fibroblast activation protein (FAP) (Zhang et al., 2026). These small discovery studies support compartment-specific investigation but do not establish the distribution of functional IL-11 responses across all strictures.

### 5.3 Imaging and molecular sampling

Magnetic resonance enterography (MRE), computed tomography enterography and intestinal ultrasound (IUS) capture different aspects of a transmurally remodeled bowel segment. Their relation to histology is imperfect, especially when inflammation and fibrosis coexist. MRE is widely used for stricture characterization, while elastography and advanced MRI sequences remain promising but incompletely standardized tools (Baumgart et al., 2015; Zhong et al., 2020; Zhang et al., 2019). International consensus defines a small-bowel stricture by the combination of wall thickening, luminal narrowing and pre-stenotic dilation; the IUS guidance further specifies response dimensions including stricture length, narrowing, dilation and motility abnormalities (Rieder et al., 2018; Lu et al., 2024).

Imaging should define the lesion and follow its structure over time. Mucosal biopsies and blood provide accessible molecular measurements, while clinically obtained full-thickness tissue can test their relationship to deeper pathology. Sampling should identify the bowel segment, layer and inflammatory activity. Figure 2 summarizes these anatomical compartments and contrasts acute injury with persistent stromal activation; cellular positions are schematic.

**FIGURE 2:**
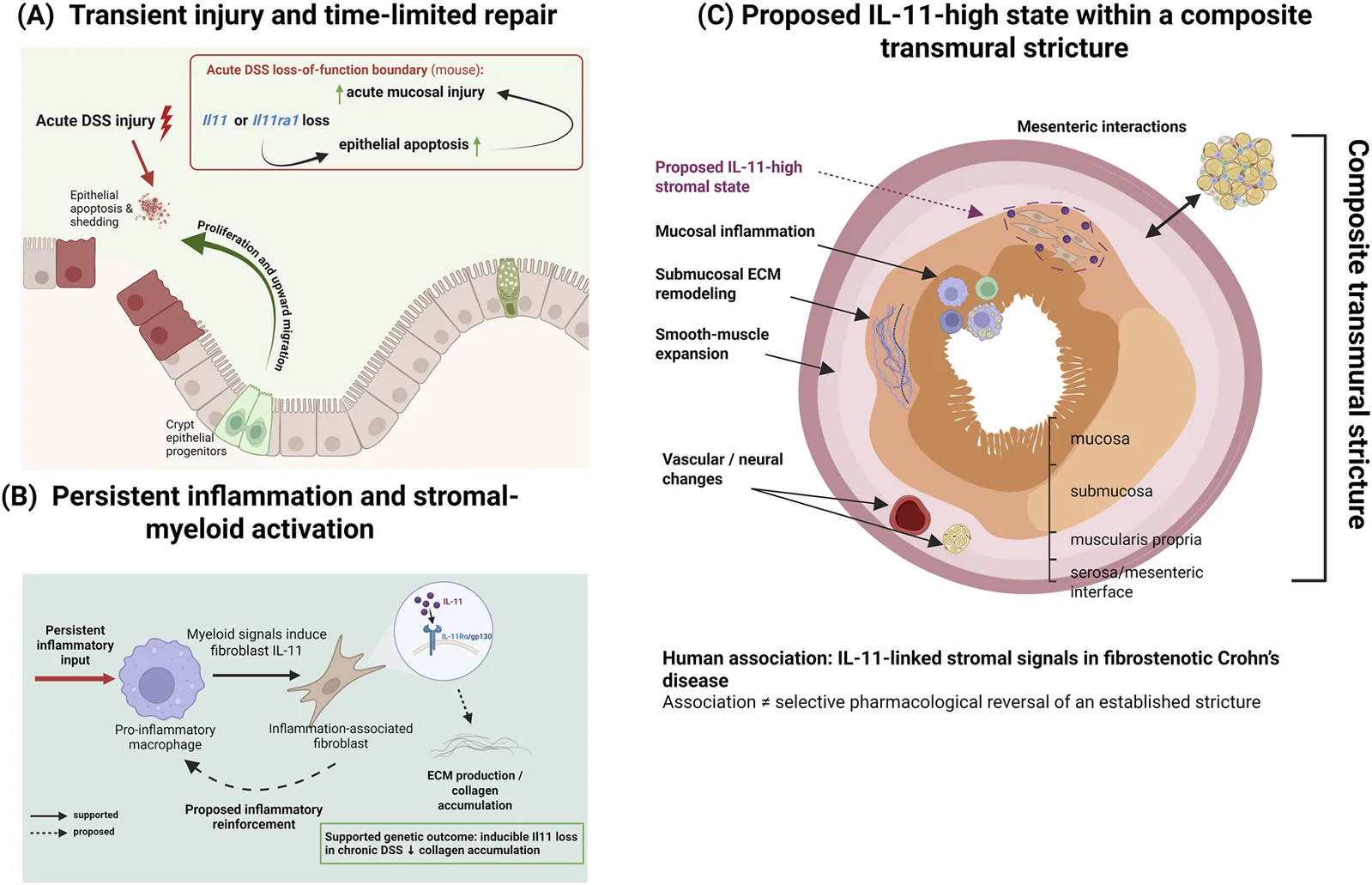
IL-11 across intestinal injury stages and bowel-wall compartments. Conceptual schematic. **(A)** In acute dextran sulfate sodium (DSS) colitis, loss of *Il11* or *Il11ra1* increases epithelial apoptosis and mucosal injury (Nishina et al., 2023). The responding cell remains unresolved; epithelial restitution illustrates repair without assigning an IL-11-responsive cell. **(B)** Proinflammatory macrophage signals can induce IL-11 production by inflammation-associated fibroblasts (Pokatayev et al., 2026). Only in **(B)** solid arrows denote supported relationships in the cited model; dashed arrows denote proposed fibroblast-autocrine signaling and inflammatory reinforcement. The autocrine route is shown as a conceptual link, without specifying receptor-complex topology. The separate genetic outcome reports reduced collagen accumulation after inducible *Il11* loss during chronic DSS. **(C)** The composite lesion includes mucosal inflammation, submucosal extracellular-matrix (ECM) remodeling, muscularis propria expansion and vascular, neural and mesenteric components. Layer dimensions and cellular positions are schematic, not an empirical map of IL-11-dependent populations. The mesenteric interaction is not asserted to be IL-11-specific. Human IL-11 associations do not establish pharmacological reversal of an existing stricture (Tan et al., 2025).

## 6 Intestinal experiments and human IL-11 studies

### 6.1 Experimental evidence in acute and chronic intestinal injury

Conditional murine *Il11* overexpression produced different outcomes according to its cellular source. Smooth-muscle-targeted expression increased intestinal inflammation, muscularis thickness, collagen staining and hydroxyproline. Fibroblast-targeted expression increased inflammation and muscularis thickness, but did not significantly increase the reported collagen or hydroxyproline measurements (Lim et al., 2020). Thus, stromal *Il11* can generate bowel pathology, but these gain-of-function experiments do not show equivalent matrix effects from every stromal source or establish dependence of an existing lesion on endogenous IL-11.

Pokatayev and colleagues tested inducible *Il11* deletion in mice undergoing three cycles of DSS injury and recovery. R26-CreERT2-mediated deletion was induced after each DSS cycle and was not fibroblast-specific. Compared with tamoxifen-treated floxed controls without Cre, deletion reduced colonic collagen-positive area, hydroxyproline and profibrotic transcripts, while inflammatory trajectories were broadly similar (Pokatayev et al., 2026). The study also identified a circuit in which proinflammatory macrophages induce IL-11 production by inflammation-associated fibroblasts. These findings establish a genetic contribution to collagen accumulation during chronic colitis. They do not test selective drug treatment after a fixed stricture has been demonstrated.

Acute DSS experiments showed a different effect. *Il11* or *Il11ra1* deficiency increased epithelial apoptosis and mucosal injury compared with control mice (Nishina et al., 2023). The responsible responding cells remain unresolved. Whole-animal deficiency therefore cannot establish an epithelial cell-autonomous mechanism or predict the safety of adult antibody treatment.

In summary, acute epithelial injury provides evidence that endogenous IL-11 can limit damage; chronic DSS inflammation provides evidence that *Il11* contributes to collagen accumulation; and established human fibrosis remains an untested setting for selective inhibition (Nishina et al., 2023; Pokatayev et al., 2026). These findings compare different models and interventions. Repair and matrix deposition can coexist within one bowel segment, so their balance need not follow a fixed temporal switch. Table 1 organizes the studies by evidence class and the highest inference supported by each design.

**TABLE 1:** Evidence classes and supported inferences for IL-11 in fibrostenotic Crohn’s disease.

Study and evidence class	Species and model or cohort	Intervention and comparator	Results and direction	Highest supported inference and boundary
Tan et al., 2025 Human association	Humans; surgical tissue and public datasets; serum n = 63; surgery subgroup n = 19	Observational: strictured/non-strictured tissue and disease-behavior groups; serum high/low at subgroup median	Tissue *IL11* RNA and IL-11 protein increased; serum higher in stricturing/penetrating disease; high serum associated with later surgery	Human tissue and serum associations; exploratory serum–surgery relationship in 19 patients, not a validated treatment-response predictor
Li et al., 2026 Human association	Human IBD public datasets; independent Crohn’s IHC samples n = 36	Observational anti-TNF non-responders *versus* responders	*IL11*-expressing fibroblast signal enriched; tissue IL-11 staining higher in 23 non-responders than 13 responders	Association with anti-TNF non-response; univariable analysis and heterogeneous IBD datasets do not establish IL-11-dependent strictures
Koplev et al., 2026 Human spatial evidence	Human full-thickness Crohn’s bowel; single-cell and spatial profiling	Observed mucosal ulcer and submucosal fibrotic niches; no IL-11 intervention	Inflammatory fibroblasts enriched in ulcers; distinct contractile stromal populations in fibrosis; *IL11* expression highest in fibroblasts, particularly inflammatory subsets	Spatially distinct stromal programs and *IL11* expression; subset-specific receptor activity and drug sensitivity remain unresolved
Pokatayev et al., 2026 Intestinal loss of function	Mice; three cycles of 2% DSS (7 days) and water (7 days); day 42	*Il11* floxed/R26-CreERT2 deletion after each DSS cycle *versus* tamoxifen-treated floxed controls without Cre	Colonic collagen area, hydroxyproline and collagen transcripts decreased; inflammatory trajectories broadly similar	Model-specific genetic contribution to chronic collagen accumulation; deletion is not fibroblast-specific and does not test drug reversal of a fixed stricture
Nishina et al., 2023 Intestinal loss of function	Mice; acute 1.5% DSS for 5 days followed by water	Global *Il11* or *Il11ra1* deficiency *versus* corresponding wild-type controls	Weight loss, colonic injury and epithelial apoptosis increased; colon length decreased	Endogenous protection during acute injury; whole-animal deficiency leaves the responding cell unresolved
Lim et al., 2020: smooth muscle Intestinal gain of function	Mice; Myh11-CreERT2/Rosa26-*Il11*; day 14	Induced murine *Il11* overexpression *versus* vehicle; Cre-only tamoxifen controls also assessed	Inflammation, muscle thickness, collagen staining and hydroxyproline increased	Sufficiency for fibro-inflammatory bowel pathology in an overexpression model; endogenous dependency remains untested
Lim et al., 2020: fibroblasts Intestinal gain of function	Mice; Col1a2-CreER/Rosa26-*Il11*; day 21	Induced murine *Il11* overexpression *versus* tamoxifen-treated wild-type littermates	Inflammation and muscle thickness increased; collagen staining, hydroxyproline and ECM transcripts not significantly changed	Inflammatory and muscular effects without a significant increase in matrix endpoints; sufficiency depends on cellular source and outcome
Sands et al., 1999 Human agonist trial	Humans; active Crohn’s disease; n = 76	Subcutaneous rhIL-11 dose/schedule groups for 3 weeks *versus* placebo	Exploratory response: 33%–42% in the 16 μg/kg/week groups *versus* 7% with placebo	Exploratory inflammatory response in small dose groups; no stricture endpoint
Sands et al., 2002 Human agonist trial	Humans; active Crohn’s disease; 148 evaluable participants	15 μg/kg weekly or 7.5 μg/kg twice weekly for 6 weeks *versus* placebo	Weekly-arm remission: 36.7% *versus* 16.3%; primary continuous CDAI endpoint not significant; twice-weekly signal not significant	Schedule-dependent remission signal with a non-significant primary continuous endpoint; no stricture endpoint
Herrlinger et al., 2006 Human agonist trial	Humans; active Crohn’s disease; 51 treated participants	rhIL-11 1 mg/week *versus* prednisolone starting at 60 mg/day	Remission at week 4: 4% *versus* 46%; week 6: 19% *versus* 50%	Inferior remission induction to prednisolone in this trial; does not test selective inhibition
Nayar et al., 2021 Adjacent mechanistic evidence	Human NOD2-risk Crohn’s samples, *ex vivo* systems and zebrafish injury	Genotype-associated comparisons and gp130-directed probes, including bazedoxifene, *versus* controls	Myeloid–stromal activation associated with NOD2 risk; gp130-directed intervention reduced the experimental phenotype	Disease-relevant myeloid–stromal pathway perturbation; gp130 inhibition is not IL-11-selective
Chen et al., 2025a: TINAGL1 Adjacent mechanistic evidence	Human mesenteric samples: 15 Crohn’s, 15 cancer controls; fibroblasts; mice with repeated DNBS	Disease-associated exosomes or recombinant TINAGL1 *versus* control preparations	Fibroblast matrix outputs and experimental intestinal fibrosis increased	Mesenteric signals can increase matrix outputs; selective TINAGL1 necessity and a direct IL-11 connection remain untested
Chen et al., 2025b: PRRX1 Adjacent delivery evidence	Human paired ileal samples from 4 patients; mice with repeated DNBS	Fibroblast *Prrx1* deletion *versus* controls; intrarectal PRRX1 hydrogel *versus* vehicle/MBP controls	Deletion reduced fibrosis; PRRX1 delivery increased FAP, collagen and histological fibrosis	Local manipulation of a stromal program; PRRX1 delivery is profibrotic, not IL-11 blockade or an antifibrotic treatment

Evidence classes distinguish human association, intestinal perturbation, historical agonist trials and adjacent mechanistic or delivery studies; they are not a numerical quality score. The final column states the highest inference supported within each design. Effect sizes are included when interpretable; otherwise the reported direction is specified. Supplementary Table S3 provides model and source details. CDAI, Crohn’s Disease Activity Index; rhIL-11, recombinant human interleukin-11; DSS, dextran sulfate sodium; DNBS, dinitrobenzene sulfonic acid; ECM, extracellular matrix; IBD, inflammatory bowel disease; IHC, immunohistochemistry; UC, ulcerative colitis; FAP, fibroblast activation protein; MBP, maltose-binding protein.

Nayar and colleagues identified an activated myeloid–stromal niche in individuals with NOD2 risk alleles and a gp130/STAT3-associated program that could be modified by bazedoxifene in a zebrafish injury model (Nayar et al., 2021). This offers evidence for disease-relevant stromal–myeloid crosstalk, but does not identify IL-11 as the sole relevant gp130 ligand or validate bazedoxifene or gp130 blockade as selective anti-fibrotic therapy for Crohn’s disease.

### 6.2 Human tissue and serum evidence

Tan and colleagues reported increased *IL11* transcripts and IL-11 protein in fibrostenotic Crohn’s tissue. Immunostaining localized IL-11 protein to COL1A1-positive cells, while single-cell RNA data placed *IL11RA* transcripts predominantly in fibroblasts. Recombinant human IL-11 increased matrix proteins in primary intestinal fibroblasts. Separately, a serum cohort comprised 63 patients: 27 with non-stricturing, non-penetrating disease, 18 with stricturing disease and 18 with penetrating disease. Serum IL-11 was higher in the latter two groups. An exploratory 19-patient subgroup linked higher serum IL-11, divided at that subgroup’s median, to subsequent surgery (Tan et al., 2025).

Surgical samples preferentially represent severe structural disease, and surgery depends on obstruction, treatment history and clinical decision-making. Prospective external validation is needed to establish the prognostic value of the serum association; sensitivity to IL-11 inhibition requires an intervention study.

### 6.3 *IL11*-expressing fibroblasts and anti-TNF response

Li and colleagues integrated public bulk and single-cell datasets and reported enrichment of *IL11*-expressing fibroblasts in anti-TNF non-responders. In a separate set of pretreatment intestinal samples from 36 patients with Crohn’s disease, IL-11 immunostaining was higher in 23 subsequent non-responders than in 13 responders (Li et al., 2026). The outcome analysis used univariable regression and did not establish independent prediction. Prior Crohn’s single-cell work identified a stromal-containing cellular module associated with anti-TNF failure in independent ileal cohorts (Martin et al., 2019). Genetic-expression studies and ulcerative colitis atlases also link stromal programs to inflammation or treatment response (Medrano et al., 2015; Smillie et al., 2019; Kinchen et al., 2018).

Anti-TNF non-response can reflect inadequate exposure, immunogenicity, persistent inflammatory pathways or symptoms caused by structural damage. The integrated cohorts used heterogeneous response definitions and included ulcerative colitis datasets. Their fibroblast associations therefore require validation in prospectively phenotyped Crohn’s strictures. They do not establish that IL-11 causes anti-TNF failure or predicts benefit from IL-11 blockade.

### 6.4 Fibroblast heterogeneity and potential treatment response

Fibroblast populations may differ in their production of IL-11 and their response to it. Human studies identify *IL11*-expressing inflammatory fibroblasts, whereas subset-specific surface IL-11Rα abundance and functional sensitivity remain incompletely defined. The responses observed in primary intestinal fibroblasts provide a starting point for testing defined subsets (Tan et al., 2025). Ligand-producing and responding cells therefore need to be studied separately.

Full-thickness spatial profiling further separates inflammatory from other stromal programs. Koplev et al. mapped COL7A1-high inflammatory fibroblasts to immune-rich mucosal ulcers, whereas GREM1-high fibrotic muscle and TNC-high myofibroblasts predominated in adjacent, relatively immune-depleted submucosal fibrosis. Fibroblasts, particularly inflammatory fibroblasts, showed high *IL11* expression (Koplev et al., 2026). These findings caution against equating increased *IL11* with all fibrotic tissue. Therapeutic effects may therefore vary across compartments: IL-11-dependent fibroblast activity could respond to blockade, whereas established matrix or contractile remodeling could persist. Whether COL7A1-high, GREM1-high or TNC-high populations differ in responsiveness remains unknown. Spatially resolved IL-11Rα protein measurements, signaling assays and selective blockade experiments are needed to identify responsive populations.

### 6.5 Research definition of an IL-11-high stromal phenotype

The term ‘IL-11-high’ requires a stated analyte, assay and comparator. Increased *IL11* transcripts in a fibroblast subset, increased tissue IL-11 protein and increased serum IL-11 are not interchangeable definitions. A future tissue classifier would require a fixed gene set or protein assay, anatomical localization and an externally tested threshold. Analyses must distinguish increased expression per cell from increased fibroblast abundance and generic inflammation. Supplementary Table S1 specifies these development requirements; a clinical scoring system has yet to be validated.

## 7 Biomarker development and target validation

No sample type currently provides a validated clinical test for an IL-11-dependent stricture. The most informative material for defining a stromal phenotype is paired, anatomically annotated full-thickness tissue obtained during clinically indicated surgery. It permits localization across mucosa, submucosa and muscularis propria, but cannot serve as a routine longitudinal test. Blood is more practical for serial monitoring, provided paired tissue and imaging studies establish what its IL-11 measurements represent.

Mucosal biopsies permit repeat measurement of local transcripts and protein but may miss the deep-wall process driving narrowing. Imaging-associated molecular signatures could combine structural assessment with accessible sampling, although these combinations remain exploratory. For serum, prospective validation must address assay reproducibility, disease activity and non-intestinal sources. For tissue, the priorities are cell and layer localization, reproducibility across sites, and separation of per-cell expression from stromal abundance.

Clinical validation depends on intended use. Diagnostic studies must compare molecular measurements with standardized stricture assessment. Prognostic studies must test future progression or intervention after accounting for anatomy, inflammation, prior surgery and treatment. A predictive biomarker requires evidence that biomarker status modifies the effect of randomized IL-11-targeted treatment. Pharmacodynamic studies should link a measurement change to drug exposure and biological response (Steiner et al., 2022). A marker of higher surgical risk is not automatically a marker of greater treatment benefit.

Pre-analytical handling and drug interference can distort apparent biological differences. Serum assays should specify calibration, free *versus* complexed ligand, storage and antibody interference. Tissue assays should record sampling depth, segment, cellularity and RNA quality. Thresholds should be fixed before external validation, with calibration and clinical utility assessed alongside discrimination.

### 7.1 Experimental requirements for target validation

No single model reproduces the full fibromuscular and obstructive phenotype of a Crohn’s stricture. Chronic DSS permits repeated injury and collagen accumulation but may not produce durable focal stenosis. Human explants and multicellular cultures preserve relevant cell interactions but cannot reproduce whole-bowel mechanics. Complementary systems are therefore needed to evaluate tissue effects and their functional consequences.

The key experiment is selective inhibition initiated after structural disease is documented. An intestinal *in vivo* model should measure target engagement, collagen, smooth-muscle changes and bowel function while assessing inflammation separately. Human tissue or fibroblast–immune–epithelial cultures should test exposure, matrix responses, viability and epithelial restitution. Species-matched pharmacology and appropriate genetic controls are required to interpret either benefit or a null result.

## 8 Pharmacological strategies and safety

### 8.1 Ligand, receptor and downstream interventions

Neutralizing IL-11 offers direct ligand selectivity and potentially interpretable free-ligand assays, provided the assay accounts for drug-bound ligand. IL-11Rα blockade may intercept receptor-proximal signaling but can differ in occupancy, internalization and tissue distribution. Engineered competitive antagonists or decoy ligands may permit distinct exposure profiles but require unusually rigorous partial-agonism testing because receptor binding without productive signaling is the intended mechanism. A long-acting antagonist has shown preclinical renal anti-fibrotic activity, demonstrating modality feasibility rather than intestinal efficacy (Pang et al., 2025).

Broad gp130 or ERK/JAK/STAT inhibition can serve as a mechanistic comparator. An effect confined to these interventions could arise through another cytokine or pathway. Selective ligand and receptor inhibition should therefore be tested separately, with their own exposure-response measurements.

Studies in lung, liver, kidney, heart and pancreas support testing IL-11 inhibition in the intestine. Selected genetic or antibody interventions reduced matrix accumulation, tissue injury or inflammation in these systems (Ng et al., 2019; Ng et al., 2020; Widjaja et al., 2019; Lim et al., 2021; Widjaja et al., 2022a; Widjaja et al., 2022b; Effenberger et al., 2023; Ng et al., 2022). Their differing endpoints and exposure requirements preclude direct extrapolation to Crohn’s strictures.

Local delivery can provide a means of testing stromal programs. Chen et al. used PEGylated black-phosphorus–alginate hydrogels to deliver recombinant PRRX1 intrarectally during repeated DNBS injury. Fibroblast-specific *Prrx1* deletion reduced fibrosis, whereas PRRX1 delivery increased FAP expression, collagen deposition and histological fibrosis (Chen et al., 2025b). The experiment demonstrates local delivery of a profibrotic payload. An IL-11-inhibitory application would require separate validation of the payload, deep-wall exposure, cell specificity and long-term safety.

### 8.2 Early clinical development outside inflammatory bowel disease

A sponsor-led report describes LASN01, an IL-11Rα-blocking antibody, in early studies outside inflammatory bowel disease (IBD). It reports systemic PK/PD and preliminary adult tolerability, including exposure up to 48 weeks (King et al., 2026). In the phase 2 thyroid eye disease study, 17 participants received LASN01 and nine received placebo. Clinical activity score response favored LASN01, but proptosis response did not. The small cohorts and disease-specific endpoints limit extrapolation to intestinal disease.

ClinicalTrials.gov entries document the designs and development status of the LASN01 studies NCT05331300 and NCT06226545 and the pulmonary-fibrosis anti-IL-11 program NCT07036523 (ClinicalTrials.gov, 2026a; ClinicalTrials.gov, 2026b; ClinicalTrials.gov, 2026c). Efficacy in Crohn’s strictures and safety during severe intestinal ulceration remain untested. Table 2 summarizes the intervention classes and remaining intestinal-development requirements.

**TABLE 2:** Candidate intervention strategies and requirements for a Crohn’s program.

Strategy	Potential advantage	Key PK/PD issue	Current evidence status	Essential evidence before Crohn’s advancement
Neutralizing anti-IL-11 antibody	Ligand-selective intervention	Free *versus* total IL-11; tissue penetration; complex-mediated clearance	Human IPF development; no IBD efficacy evidence	Gut target engagement, established-fibrosis intervention and Crohn’s safety
Anti-IL-11Rα antibody	Receptor-proximal blockade	Cell-specific occupancy, internalization and receptor expression	Small adult LASN01 studies reported systemic PK/PD and preliminary tolerability. Thyroid eye disease activity favored treatment, but proptosis response did not; no IBD efficacy evidence (King et al., 2026)	Human intestinal occupancy, post-establishment pharmacology, multicellular safety and Crohn’s efficacy testing
Engineered IL-11 antagonist/decoy	Potentially tunable size and exposure	Partial agonism and species-specific activity	Preclinical renal example	Species-matched agonism/antagonism assays and receptor-null controls
gp130-directed intervention	Can test a stromal–myeloid signaling neighborhood	Shared-cytokine effects and broad toxicity	Experimental Crohn’s-relevant evidence	Demonstrate any benefit is IL-11 dependent
ERK/JAK/STAT-directed intervention	Mechanistic comparator or combination probe	Pleiotropy; pSTAT3 is non-specific	Existing drug classes	Use after, not instead of, IL-11-selective validation

PK/PD, pharmacokinetics/pharmacodynamics; IPF, idiopathic pulmonary fibrosis; IBD, inflammatory bowel disease.

### 8.3 Treatment timing and intestinal safety

Biallelic *IL11RA* variants cause developmental craniosynostosis syndromes (Keupp et al., 2013; Ahmad et al., 2023). Congenital deficiency differs from reversible adult drug exposure, but identifies developmental and skeletal domains requiring consideration. Historical agonist exposure additionally supports hematologic monitoring. These observations generate safety questions rather than proving that adult inhibition will produce the same outcomes.

Treatment timing may determine the balance between suppressing matrix production and interfering with repair. The acute and chronic model findings raise a timing hypothesis: an initial study could favor patients with controlled mucosal inflammation and persistent, predominantly fibrotic narrowing rather than severe active ulceration (Nishina et al., 2023; Pokatayev et al., 2026). This selection strategy requires safety testing; persistent fibrosis can coexist with active repair, and the reparative role of IL-11 in humans remains uncertain.

Early studies should monitor endoscopic ulceration, inflammatory markers, wound healing, infections and hematologic effects alongside exposure. Pregnancy, skeletal outcomes and immunogenicity require appropriate surveillance. Prespecified interruption criteria should address new ulceration, poor healing or infection before prolonged treatment is evaluated.

Background biologic or small-molecule therapy must be recorded because combination treatment can alter infection risk, exposure and inflammatory outcomes. Worsening obstruction, abscess or penetrating complications require timely standard intervention. Murine tumor studies identify additional surveillance questions but do not justify claims of cancer prevention by an intestinal antifibrotic program (Nishina et al., 2021; Huynh et al., 2021; Xiong et al., 2023).

## 9 Considerations for an early intestinal clinical study

An early Crohn’s study should follow adequate intestinal pharmacology and repair-safety experiments. Clinical remission alone would be an inadequate endpoint because symptoms can change through inflammation, edema or structural narrowing. The initial objectives should be safety, intestinal exposure and target engagement, with structural outcomes considered exploratory.

A randomized add-on study could enroll adults with non-emergent ileal or ileocolonic strictures receiving stable, optimized anti-inflammatory care. Central imaging should document stricture location, length, narrowing, upstream dilation and inflammatory activity. Prior surgery, anastomotic status and background therapy should be recorded. Patients requiring urgent dilation or surgery would not be appropriate for a prolonged experimental-treatment window.

Sample timing should separate early target engagement from later changes in tissue and structure. Safe mucosal biopsies and serial blood samples can provide exploratory molecular measurements. Clinically indicated surgical tissue can support anatomical validation. Blinded, standardized MRE or IUS should follow the same lesion, with inflammation assessed independently.

Exploratory outcomes could include luminal caliber, upstream dilation, wall thickness, motility and obstruction-related intervention. These imaging features are not validated surrogate endpoints for IL-11-targeted therapy. Consensus work supports collecting clinically meaningful outcomes such as dilation and surgery, while recognizing their dependence on management decisions (Rieder et al., 2018; Danese et al., 2018).

Interpretation should distinguish inadequate tissue exposure or insufficient follow-up from a biological null result. Structural stabilization, defined as less progression than in the control group, must be distinguished from regression relative to baseline. Failure to demonstrate regression would not exclude an effect on ongoing fibrogenesis or progression; it would leave the stronger claim of reversible dependence unproven. Within a reproducibly defined baseline stromal phenotype, confirmed target engagement without a matrix or functional benefit over an appropriate observation period would weaken the dependency hypothesis at the tested stage and exposure. Concordant tissue and structural improvement would justify a larger trial, provided it cannot be explained solely by reduced inflammation. These alternatives should be specified before unblinding.

## 10 Discussion and conclusions

Current evidence supports a role for *Il11* in collagen accumulation during chronic experimental colitis and suggests associations with human fibrostenosis. Acute-deficiency studies also identify a possible role in mucosal repair. Together, these findings justify investigation while preserving uncertainty about benefit and harm in humans.

The central uncertainty is whether an IL-11-high stromal state identifies an ongoing, pharmacologically reversible dependency within an established stricture or mainly marks advanced remodeling. Resolving it requires identification of responding cell populations and the disease contexts in which their activity remains modifiable. The heterogeneous, author-selected evidence assembled here supports this question but cannot determine which interpretation applies to an individual lesion.

The next priority is selective inhibition after an intestinal structural lesion is documented, with verified tissue exposure and separate structural, inflammatory and repair endpoints. Prospective human studies should establish biomarker reproducibility and test whether the baseline stromal state predicts treatment response. Together, these studies would determine whether selective IL-11 inhibition can improve established bowel-wall structure and function.
