# Supplementary material for: IL-11 in fibrostenotic Crohn’s disease: historical agonism, the selective-inhibition hypothesis, and evidence thresholds for translation
Source: Front Pharmacol. 2026 Oct 16;17:1951110. doi: 10.3389/fphar.2026.1951110 (PMC13720363; doi:10.3389/fphar.2026.1951110)
Supplement: Supplementary file 1 [file Supplementaryfile1.docx]

**Supplementary Material for the *IL11* Crohn’s Disease Review**

# Supplementary Methods

This critical narrative review used author-led selection without pooled effect estimates or formal risk-of-bias scores.

## **Sources and dates**

PubMed was searched from inception through 28 July 2026; ClinicalTrials.gov was checked on the same date. Crossref verified bibliographic metadata and DOIs. Reference lists of foundational trials, receptor-pharmacology papers, consensus statements and primary intestinal studies were hand-searched. Author and exact-title searches verified primary reports.

## **Search queries**

The following PubMed-style query was used as the main discovery search:

(("interleukin-11"[Title/Abstract] OR "IL-11"[Title/Abstract] OR IL11[Title/Abstract]

OR IL11RA[Title/Abstract] OR "IL-11 receptor"[Title/Abstract])

AND

(Crohn*[Title/Abstract] OR "inflammatory bowel disease"[Title/Abstract]

OR IBD[Title/Abstract] OR intestinal[Title/Abstract] OR bowel[Title/Abstract])

AND

(fibrosis[Title/Abstract] OR fibrostenosis[Title/Abstract] OR strictur*[Title/Abstract]

OR stenosis[Title/Abstract] OR stromal[Title/Abstract] OR fibroblast*[Title/Abstract]))

Targeted queries addressed historical agonism, receptor pharmacology, intestinal injury models, stricture assessment, treatment resistance and clinical development:

("recombinant human interleukin-11" OR rhIL-11) AND (Crohn* OR colitis OR mucositis)

(IL11 OR IL11RA) AND (gp130 OR IL6ST OR receptor OR binding OR species OR mouse OR human)

(Crohn* AND strictur*) AND (MRI OR MRE OR ultrasound OR elastography OR histology OR "smooth muscle")

(Crohn* OR IBD) AND ("anti-TNF" OR non-response OR resistance OR stromal OR "single-cell")

(IL11 OR IL11RA) AND (DSS OR colitis OR macrophage OR fibroblast OR "conditional deletion")

(IL11 OR IL11RA) AND (antibody OR antagonist OR LASN01 OR "thyroid eye disease" OR fibrosis OR safety OR cancer)

## Targeted literature update on 13 September 2026

Targeted searches on 13 September 2026 addressed mesenteric TINAGL1, local PRRX1 delivery, fibroblast heterogeneity and spatial profiling of Crohn’s tissue. PubMed, PMC/Europe PMC and publisher full texts were checked by concept, author and exact title. Broader search and registry dates were unchanged.

Targeted concepts included: Crohn’s + TINAGL1 + exosomes; intestinal fibrosis + PRRX1 + hydrogel; Crohn’s + *IL11* + fibroblast + single-cell/spatial; and exact titles and DOIs of the relevant primary reports.

## **Study selection**

Eligible English-language full articles addressed Crohn’s trials, intestinal tissue or experiments, receptor/species pharmacology, cross-organ pharmacology, safety, or stricture assessment and endpoints. Peer-reviewed online-first articles with stable journal records and DOIs were eligible.

Title/abstract screening preceded full-text review; selection was not dual-independent. Conference-only records, unverified preprints and methodologically insufficient reports were excluded from core causal or translational support. Registry entries described development status, not efficacy.

## **Evidence classification and limitations**

Priority was given to controlled human studies, direct intestinal evidence, genetic perturbation and species/receptor-defined pharmacology. Related datasets, overlapping cohorts and related platforms were not treated as independent replication.

Studies were classified as human intestinal association, intestinal experimental evidence, receptor/species pharmacology, cross-organ plausibility, safety/development evidence, or consensus/endpoint guidance. Surgical tissue studies were assessed for selection and late-disease bias. Cross-organ studies informed tractability and safety, not Crohn’s stricture dependence.

English-language restriction, author-led selection and publication bias may limit coverage.

# Supplementary Table S1 Research requirements for an IL-11-high stromal phenotype

These requirements concern development of an exploratory tissue classifier, not a validated clinical test. Transcript, tissue-protein and serum measurements require separate definitions.

| **Requirement** | **Specification** |
| --- | --- |
| Intended use and analyte | Specify a tissue-linked pharmacodynamic or enrichment use in imaging-defined fibrostenotic Crohn’s disease. Define *IL11* RNA, tissue IL-11 protein and serum IL-11 separately. None is a validated diagnostic, prognostic or treatment-predictive test. |
| Cellular source and anatomical layer | Identify the producing cells and bowel-wall layer or mesenteric compartment. Distinguish expression per cell from fibroblast abundance. Confirm localization using spatial or cell-resolved methods. |
| Receptor competence and response | Assess *IL11RA*/*IL6ST* transcripts separately from receptor protein and functional responsiveness. Derive candidate response genes from human intestinal stimulation or selective-blockade experiments and replicate independently. Ligand production does not establish receptor competence. |
| Comparators and confounders | Account for inflammation, stromal content, ECM, muscle remodeling, intestinal segment, disease activity, prior surgery, sampling depth and treatment exposure. Test whether the signal persists beyond these factors. |
| Assay processing and threshold | Pre-specify assay units, normalization, quality filters, batch correction and missing-value handling. Derive any gene weights and threshold in training data; lock them before blinded external testing. |
| Tissue and protein confirmation | Validate antibody specificity and negative controls for IHC. Co-register anatomical markers and confirm with RNA in situ hybridization or another orthogonal assay where feasible. Distinguish RNA expression from protein positivity. |
| External and clinical validation | Use an independent centre and preferably a distinct platform. Assess reproducibility, calibration, discrimination and prospectively defined tissue/imaging outcomes. Treatment-predictive use requires a randomized treatment-by-biomarker interaction for an IL-11-directed agent. |

# Supplementary Table S2 Historical studies of IL-11 and mucosal protection

The selected studies document the historical mucosal-protection rationale and a later acute-injury genetic counterpoint. Complete references are provided in the main manuscript.

| **Study** | **Model and host species** | **IL-11 reagent and schedule** | **Injury stage / primary endpoint** | **Main result** | **Modern interpretation** |
| --- | --- | --- | --- | --- | --- |
| Du et al., 1994 | Mouse; combined chemotherapy and radiation small-intestinal injury | IL-11 administered after combination radiation/chemotherapy; the accessible abstract/metadata do not provide enough detail to verify formulation, dose, route or frequency | Acute crypt/villus injury; survival and crypt-cell proliferation | Improved survival and rapid mucosal recovery with higher crypt mitotic activity | Acute rodent mucosal-repair evidence; does not test chronic transmural fibrosis, fixed narrowing or species-matched endogenous signaling. |
| Orazi et al., 1996 | 78 C3H/HeJ mice; 5-fluorouracil followed by 7 Gy total-body irradiation | IL-11 250 μg/kg/day, divided twice daily subcutaneously, started on the irradiation day | Acute small-intestinal injury; apoptosis, crypt proliferation and villus/crypt morphology | Less crypt apoptosis, more proliferation and faster mucosal recovery | A well-defined acute injury experiment; endpoint and time course are distinct from established Crohn’s fibrostenosis. |
| Keith et al., 1994 — multi-model report | Golden Syrian hamster oral mucositis; Sprague–Dawley rat acute colonic injury; HLA-B27/β2-microglobulin transgenic Fischer 344 rat colitis | rhIL-11, twice-daily subcutaneous administration; dose-response in oral mucositis | Acute/experimental mucosal injury; gross and microscopic damage | Reduced mucositis and colonic injury in the reported models | Supports the historical mucosal-protection rationale, but combines species and models and does not measure fixed strictures or deep-wall remodeling. |
| Trepicchio et al., 1997 | Activated peritoneal macrophages | rhIL-11 exposure in vitro | Inflammatory mediator production and NF-κB activity | Reduced pro-inflammatory mediator production and NF-κB nuclear activity | Mechanistic support for an immunomodulatory hypothesis; not an intestinal structural-disease model. |
| Nishina et al., 2023 — stage-specific genetic counterpoint | *Il11*- or *Il11ra1*-deficient mice; acute DSS colitis | Endogenous-pathway loss of function; no therapeutic agonist | Acute colonic mucosal injury; epithelial apoptosis and colitis susceptibility | Genetic deficiency increased epithelial apoptosis and worsened acute DSS colitis | Modern evidence that endogenous IL-11 can support acute mucosal protection; it cannot be read as evidence for chronic stricture biology or human blockade safety. |

# Supplementary Table S3 Additional model and assay details for main Table 1

Studies follow the order of main Table 1. This table provides model, comparator, timing and assay details; results and evidence-level interpretations remain in main Table 1. Complete references are provided in the main manuscript.

| **Study** | **Model and comparator** | **Additional traceability detail** |
| --- | --- | --- |
| Tan et al., 2025 | Humans; surgical tissue and public datasets; serum n = 63; surgery subgroup n = 19 Observational: strictured/non-strictured tissue and disease-behavior groups; serum high/low at subgroup median | Serum behavior groups: B1 n=27, B2 n=18, B3 n=18. Surgery analysis: n=19, median split at 42.02 pg/mL (low n=11; high n=8). This exploratory threshold is not externally validated. Tissue ligand protein and receptor transcripts are distinct assays. |
| Li et al., 2026 | Human IBD public datasets; independent Crohn’s IHC samples n = 36 Observational anti-TNF non-responders versus responders | Pretreatment Crohn’s intestinal IHC: 23 subsequent non-responders and 13 responders; mucosal/submucosal fields. Outcome regression was univariable. Integrated discovery/validation cohorts and the UC external model test are not a prospective stricture cohort. |
| Koplev et al., 2026 | Human full-thickness Crohn’s bowel; single-cell and spatial profiling Observed mucosal ulcer and submucosal fibrotic niches; no IL-11 intervention | Figure 2b–f localizes COL7A1-high inflammatory fibroblasts to mucosal ulcers and GREM1-high muscle/TNC-high myofibroblasts to submucosal fibrosis. Extended Data Figure 5d/e supports the *IL11* expression pattern. No subset-specific IL-11Rα functional intervention. |
| Pokatayev et al., 2026 | Mice; three cycles of 2% DSS (7 days) and water (7 days); day 42 *Il11* floxed/R26-CreERT2 deletion after each DSS cycle versus tamoxifen-treated floxed controls without Cre | 2% DSS for 7 days followed by water for 7 days, repeated three times. Tamoxifen for 3 days after each DSS cycle. Day-42 assessment; chronic-DSS comparison groups reported as 17 versus 10 mice. R26-CreERT2 is not fibroblast-specific. |
| Nishina et al., 2023 | Mice; acute 1.5% DSS for 5 days followed by water Global *Il11* or *Il11ra1* deficiency versus corresponding wild-type controls | Male mice aged 9–15 weeks; 1.5% DSS for 5 days. Epithelial injury/apoptosis assessed during acute injury; colon length assessed at day 15. Genotype groups co-housed; whole-animal deficiency does not identify the responding cell. |
| Lim et al., 2020: smooth muscle | Mice; Myh11-CreERT2/Rosa26-*Il11*; day 14 Induced murine *Il11* overexpression versus vehicle; Cre-only tamoxifen controls also assessed | Myh11-CreERT2/Rosa26-*Il11*, six-week-old males; tamoxifen 50 mg/kg on days 0, 3 and 5. Day-14 assessment. Vehicle and Cre-only controls address induction-related effects. |
| Lim et al., 2020: fibroblasts | Mice; Col1a2-CreER/Rosa26-*Il11*; day 21 Induced murine *Il11* overexpression versus tamoxifen-treated wild-type littermates | Col1a2-CreER/Rosa26-*Il11*, six-week-old mice of both sexes; tamoxifen 50 mg/kg for 10 days, assessment at day 21. Fibrosis endpoints were not significantly increased despite inflammation and muscle thickening. |
| Sands et al., 1999 | Humans; active Crohn’s disease; n = 76 Subcutaneous rhIL-11 dose/schedule groups for 3 weeks versus placebo | rhIL-11 dose groups: 5, 16 or 40 μg/kg/week, split into two or five injections weekly. The 16 μg/kg/week groups had response rates of 33% and 42%, respectively; placebo 7%. |
| Sands et al., 2002 | Humans; active Crohn’s disease; 148 evaluable participants 15 μg/kg weekly or 7.5 μg/kg twice weekly for 6 weeks versus placebo | Placebo n=49; 15 μg/kg once weekly n=49; 7.5 μg/kg twice weekly n=50; six weeks. Primary continuous CDAI endpoint was not significant. |
| Herrlinger et al., 2006 | Humans; active Crohn’s disease; 51 treated participants rhIL-11 1 mg/week versus prednisolone starting at 60 mg/day | rhIL-11 n=27 versus prednisolone n=24; twelve-week trial. Reported remission comparisons at weeks 4 and 6 concern induction, not structural disease. |
| Nayar et al., 2021 | Human NOD2-risk Crohn’s samples, ex vivo systems and zebrafish injury Genotype-associated comparisons and gp130-directed probes, including bazedoxifene, versus controls | The gp130/STAT3-associated myeloid–stromal niche was investigated using human material and experimental systems. gp130-directed intervention is not an IL-11-selective perturbation; no claim of established-stricture reversal is made. |
| Chen et al., 2025a: TINAGL1 | Human mesenteric samples: 15 Crohn’s, 15 cancer controls; fibroblasts; mice with repeated DNBS Disease-associated exosomes or recombinant TINAGL1 versus control preparations | Human controls were colorectal-cancer surgical specimens, not healthy volunteers. Repeated DNBS over 6 weeks; disease exosomes intravenously or recombinant TINAGL1 in oral hydrogel during injury; main mouse groups n=6. Interaction/signaling assays implicate SMAD4 but do not establish necessity. |
| Chen et al., 2025b: PRRX1 | Human paired ileal samples from 4 patients; mice with repeated DNBS Fibroblast *Prrx1* deletion versus controls; intrarectal PRRX1 hydrogel versus vehicle/MBP controls | Four paired strictured/non-strictured ileal donors for scRNA-seq. Col1a2-CreERT2/*Prrx1* deletion induced before 6-week DNBS. PRRX1 hydrogel given intrarectally during injury; vehicle and MBP controls, main groups n=6. Delivery increased, rather than reduced, fibrosis. |

Abbreviations: B1, non-stricturing and non-penetrating Crohn’s disease; B2, stricturing disease; B3, penetrating disease; CDAI, Crohn’s Disease Activity Index; DSS, dextran sulfate sodium; DNBS, dinitrobenzene sulfonic acid; ECM, extracellular matrix; IBD, inflammatory bowel disease; IHC, immunohistochemistry; MBP, maltose-binding protein; scRNA-seq, single-cell RNA sequencing; UC, ulcerative colitis.
